# The Nanomaterial Data Curation Initiative: A collaborative approach to assessing, evaluating, and advancing the state of the field

**DOI:** 10.3762/bjnano.6.179

**Published:** 2015-08-18

**Authors:** Christine Ogilvie Hendren, Christina M Powers, Mark D Hoover, Stacey L Harper

**Affiliations:** 1 Center for the Environmental Implications of NanoTechnology, Duke University, Durham, NC, USA; 2 National Center for Environmental Assessment, Office of Research and Development, U.S. Environmental Protection Agency, RTP, NC, USA; 3 current affiliation: Office of Transportation and Air Quality, Office of Air and Radiation, U.S. EPA, Ann Arbor, MI, USA; 4 National Institute for Occupational Safety and Health, Morgantown, WV, USA; 5 Department of Environmental and Molecular Toxicology, Oregon State University, Corvallis, OR, USA; 6 School of Chemical, Biological and Environmental Engineering, Oregon State University, Corvallis, OR, USA

**Keywords:** curation, data integration, interoperability, nanoinformatics, nanomaterials

## Abstract

The Nanomaterial Data Curation Initiative (NDCI), a project of the National Cancer Informatics Program Nanotechnology Working Group (NCIP NanoWG), explores the critical aspect of data curation within the development of informatics approaches to understanding nanomaterial behavior. Data repositories and tools for integrating and interrogating complex nanomaterial datasets are gaining widespread interest, with multiple projects now appearing in the US and the EU. Even in these early stages of development, a single common aspect shared across all nanoinformatics resources is that data must be curated into them. Through exploration of sub-topics related to all activities necessary to enable, execute, and improve the curation process, the NDCI will provide a substantive analysis of nanomaterial data curation itself, as well as a platform for multiple other important discussions to advance the field of nanoinformatics. This article outlines the NDCI project and lays the foundation for a series of papers on nanomaterial data curation. The NDCI purpose is to: 1) present and evaluate the current state of nanomaterial data curation across the field on multiple specific data curation topics, 2) propose ways to leverage and advance progress for both individual efforts and the nanomaterial data community as a whole, and 3) provide opportunities for similar publication series on the details of the interactive needs and workflows of data customers, data creators, and data analysts. Initial responses from stakeholder liaisons throughout the nanoinformatics community reveal a shared view that it will be critical to focus on integration of datasets with specific orientation toward the purposes for which the individual resources were created, as well as the purpose for integrating multiple resources. Early acknowledgement and undertaking of complex topics such as uncertainty, reproducibility, and interoperability is proposed as an important path to addressing key challenges within the nanomaterial community, such as reducing collateral negative impacts and decreasing the time from development to market for this new class of technologies.

## Introduction

The topic of Big Data, and its promise to combine and analyze vast amounts of information to produce new knowledge, has gained widespread interest across many fields and in popular science literature today. The bioinformatics community provides a concrete illustration of the value that mechanisms for synthesizing large and disparate datasets could bring to the broader scientific community. Collaborative approaches to synthesize data add value to the scientific community in terms of a variety of parameters, including: leveraging research investments across multiple initiatives, facilitating trans-disciplinary translation of information, accelerating scientific discovery, and enabling faster risk assessment and commercialization of new technologies. These parameters are especially critical for emerging technologies, such as nanotechnology. The issues addressed in this initiative are certainly not unique to nanomaterials; in fact, they are important to chemistry, materials science and toxicology fields as a whole. However, drawing on existing experience with standards development, data handling and data integration to address viable solutions for complex data integration within the scope of nanomaterial data may serve as a specific case that could ultimately provide insights useful to broader data spheres.

### Challenges for the global development of engineered nanomaterials

Researchers and product developers around the globe are currently working toward understanding and controlling the behavior of matter at the nanoscale. Engineered nanomaterials (ENMs), typically classified as materials with at least one dimension between 1 and 100 nanometers that exhibit unique physical, biological, or chemical behavior due to their size, present both the opportunity to harness their novel properties for a wide range of applications, as well as to anticipate and mitigate potential collateral consequences (e.g., accumulation of biopersistent materials in environmental media and latent adverse health effects of a material) [1–2]. Because understanding the behavior of nanomaterials of natural or incidental origin is a critical aspect of investigating the impacts of nanomaterials that are engineered, data are being gathered on all classes of these materials; therefore, throughout the paper we refer to “nanomaterials” to encompass all types (i.e., natural, incidental, engineered), except in cases in which we explicitly state ENM(s). The large variety of potential nanomaterial physicochemical characteristics and applications has led to diverse and rapidly emerging data in terms of materials (both pristine and modified), their interactions in environments (both laboratory-based and natural), and across a broad spectrum of potentially relevant biological interactions. The prospect of integrating nanomaterial datasets is thus difficult in itself. Add to this the fact that protocols for fabricating, measuring and testing nanomaterials are still in the process of being developed. Moreover, nanomaterials are dynamic, often transforming dramatically upon release to the environment, or into the body. Such challenges make the process of integrating diverse nanotechnology-related datasets a seemingly intractable problem. Progress toward defining and achieving a level of “functional interoperability” of datasets, which we define as the level of sameness within a dataset that facilitates sharing and comparison for a given analytical purpose, will require a collaborative effort by the nanomaterial community (i.e., researchers, product developers, funding agencies, regulators). Specifically, community members will need to define the purposes for sharing and to develop and apply complementary approaches to collect, manage and share data in ways that can support those purposes.

### Community focus on building effective nanoinformatics approaches

The need for collaborative and dedicated attention to informatics in the nanomaterial community was a focal point of two recent National Research Council (NRC) reports on nanomaterial research progress for environment, health and safety (EHS) [3–4]. A number of efforts to begin enabling interoperability in nanomaterial datasets are already underway that draw on established data management approaches. Examples of specifically funded data repository projects include: the RTI International Nanomaterial Registry (http://www.nanomaterialregistry.org) and the National Cancer Institute (NCI) Nanotechnology Characterization Lab (http://ncl.cancer.gov). The Nanotechnology Knowledge Infrastructure (NKI), one of six signature initiatives of the National Nanotechnology Coordination Office, also provides a resource for federal agencies in the United States to work toward shared data streams (http://www.nano.gov/NSINKI). The Materials Genome Initiative (http://materialsinnovation.tms.org/genome.aspx) is a broader, but related data management effort to catalogue materials and their key characteristics [5].

Prior to the development of these efforts, the NCI established the National Cancer Informatics Program (NCIP) Nanotechnology Working Group (Nano WG) for nanomaterial researchers with a specific interest in informatics and computational approaches. This working group includes active membership and input from many communities (e.g., nanoEHS, commercial industry, standards community), but began with a particular emphasis on nanomedicine. From this area of emphasis, the NCIP NanoWG is well-positioned to serve as a conduit for sharing experience and best practices of the bioinformatics community with the emerging nanoinformatics community. In doing so, the NCIP NanoWG facilitates the translation of lessons learned in prior efforts to link disparate datasets and probe important community research questions; the group also leads discussions of data issues unique to the uncertainties inherent to nanomaterials and other emerging technologies that have inherent uncertainties. The NCIP NanoWG now encompasses additional stakeholder groups including industry representatives and environmental risk forecasters, all similarly interested in how the novel properties of engineered nanomaterials affect their interactions and behavior.

Since its inception, the NCIP NanoWG has supported the development of the NanoParticle Ontology (NPO) (http://www.nano-ontology.org) vocabulary standards, first published in 2011 and periodically updated. In addition, the group recently developed and published data-exchange standards along with tools to enable the use of these standards (ISA-TAB-Nano; ASTM International E2909-13) [6]. To build on these efforts, the NCIP NanoWG is now developing a shared vision for curation of data related to nanoscale materials via the broad, community-inclusive NDCI project presented here.

### A vision of nanoinformatics roles and responsibilities

The NCIP NanoWG-lead Nanomaterial Data Curation Initiative (NDCI) explores the critical aspect of data curation within the development of informatics approaches to understanding nanomaterial behavior. The following working definition (expanded from the Nanoinformatics 2020 Roadmap [7]) has been proposed [8]: “*Nanoinformatics is the science and practice of determining which information is relevant to meeting the objectives of the nanoscale science and engineering community, and then developing and implementing effective mechanisms for collecting, validating, storing, sharing, analyzing, modeling, and applying the information, and then confirming that appropriate decisions were made and that desired mission outcomes were achieved,*[…]” with additional steps in the informatics lifecycle including “[…]*conveying experience to the broader community, contributing to generalized knowledge, and updating standards and training*.” Successful nanoinformatics endeavors will apply all of the steps in the process.

In the context of the overall working definition of nanoinformatics, the roles and responsibilities of the myriad individuals who are engaged in the development and application of nanotechnology can be viewed as fitting into four categories: data *customers* (who specify the data needs for their intended purposes), data *creators* (who will develop relevant and reliable data to meet the customer needs), data *curators* (who will perform the central roles described in this NDCI work), and data *analysts* (who will develop and apply models for data analysis and interpretation that are consistent with the quality and quantity of the data and that meet customer needs). In some instances, the same individuals may perform all roles, and in the larger global reality the individuals and their roles may extend over significant distances, organizations, and time periods.

### The central role of curation

Data curation has been defined as “the active and on-going management of data through its lifecycle of interest and usefulness to scholarship, science, and education; curation activities enable data discovery and retrieval, maintain quality, add value, and provide for re-use over time” [9]. Data curation has been chosen as the focus of the collaborative initiative because of its central role in facilitating all aspects of the informatics lifecycle. Resources like those noted above that are developing to organize and analyze nanomaterial data represent efforts that can differ widely in terms of data scopes, driving goals, and development phases. Despite these potentially divergent aspects, one commonality shared across all nanoinformatics resources is that data must be *curated* into those resources.

### The purpose of this article

This article outlines the NDCI project and lays the foundation for a series of papers on nanomaterial data curation. Ultimately, through this series of papers, the NDCI will: 1) present and evaluate the current state of nanomaterial data curation across the field on multiple specific data curation topics, 2) propose ways to leverage and advance progress for both individual efforts and the nanomaterial data community as a whole, and 3) provide opportunities for similar publication series on the details of the interactive needs and workflows of data customers, data creators, and data analysts.

The specific objectives of the NDCI paper series include:

- to capture a snapshot of current nanomaterial data curation practices and issues,
- to develop recommendations for moving the nanoinformatics community toward increasingly standardized curation practices; and
- to facilitate collaborations between researchers, product developers, and others working with nanomaterials that establish and utilize common datasets for cross-boundary work (e.g., application of data from an academic institution to nanomaterial product development in industry).

In the subsequent sections below, we expand on the rationale and approach for our focus on data curation as an integral piece within the nanomaterial community’s efforts to progress towards the functional interoperability of datasets, and we conclude with an invitation for active community collaboration in these efforts.

### The NDCI focus on data curation

#### The motivation

The term nanoinformatics can encompass a vast scope and differ in meaning to different audiences. These scopes and meanings may refer to such diverse data types and uses as: catalogues of self-identified nano-enabled products on the market; efforts to derive nano-specific quantitative structure activity relationships (QSARs); or estimating environmental concentrations based on a mixture of measurements and models. The range of definitions, scopes and purposes of nanomaterial data-driven efforts is broad, but what is shared between these efforts are the needs to leverage limited resources and to understand clearly what the emerging data mean. There are many aspects to consider and optimize in moving toward a true knowledge or data commons as called for in various ways by the NRC, the NNI and the EU Nanosafety Cluster. Multiple focal areas and driving goals must be considered across the data life cycle; multiple roles exist as well, with different orientations toward the data including creators, customers, curators, and analysts. At this nascent stage in the formation of a nanoinformatics community, even in the face of so much disparity, one common aspect shared across all nanoinformatics resources is that in some form, data must be curated into them. Through exploration of sub-topics related to all activities necessary to enable, execute, and improve the curation process, it is our goal that the NDCI will provide a substantive analysis of nanomaterial data curation itself, as well as a platform for multiple other important discussions to advance the field of nanoinformatics.

Scientific data curation, a mature field within library science and a maturing sub-field of most data-driven academic domains, is increasingly a topic of interest within the nanomaterial research and associated nanoinformatics communities [10]. The methods, protocols and parameters guiding data generation within this young area of science are developing in parallel with data characterizing these novel materials, their performance, and their potential impacts. With the innumerable materials, functionalities, and complex application and implication scenarios, testing ENMs on a case-by-case basis is an intractable proposition; leveraging research investments across the community will be critical to enable the type of iterative feedback between disciplines and sectors necessary to meet the important challenges of responsibly commercializing nanotechnologies. By working together from the beginning to tackle difficult data issues including uncertainty, reproducibility, and interoperability of complex datasets, the nanoinformatics community could collaboratively address these challenges. In doing so, the community can help decrease the time from development to market and reduce collateral negative impacts of nano-enabled technologies.

The goals of this initiative are to describe the current baseline of curation practices and to develop recommendations for moving the nanoinformatics community forward. Data curation is a broad term encompassing all aspects involved with assimilating data into centralized repositories or sharable formats. Borrowed from the concept of art curation, the term “curation” is selected to signify that this process entails more than a series of data management tasks, but also includes elements of discernment and judgment inherent to this decision process. The curation practices captured through the NDCI will incorporate aspects of both reasoning and methods for curation steps including: sourcing and parsing of information into datasets; organizing data into cyberinfrastructures; formatting data for current or future interoperability; and identifying implications that commonly adopted data and meta-data formatting conventions may have on defining data quality and therefore impacting future experimental design. A goal of the NCIP NanoWG Nanomaterial Data Curation Initiative is to help establish an understanding of what a wide range of stakeholders in data curation mean when they talk about and undertake this process. In doing so, we can identify synergies and disconnects between different efforts, both of which are necessary to advance toward interoperability of large, disparate nano datasets. There are many ways to orient a discussion on the integration of tools and datasets; nano curation was selected as a focus because the process of understanding how different organizations consume and manage nanotechnology related data will require us to explicitly discuss underlying assumptions and practical approaches to individual efforts. In turn, we can better understand and communicate with the scientific community what would be required to integrate the efforts. Though we will present synthesized recommendations for moving forward, we are also committed to reporting dissenting opinion. Indeed, where disagreement can be identified, we may diagnose the root cause of disconnects between approaches to curation. This in itself will represent a useful exercise as we map out the landscape of nano data curation and determine what level of interoperability between datasets and systems will be necessary to support a range of goals across the community (e.g., developing new ENM consumer products, designing nanotherapeutics, evaluating potential toxicity of multiple nanomaterial types).

The fundamental driver underlying all the layers of the nano curation discussion is to understand: What is it that must match between materials, systems, and data fields in order to enable comparisons? This project will move through that question by probing what is meant by each part of this fundamental question: What materials? What systems? What data fields? And what comparisons? The answer to these questions, as expected, will be “it depends”. Our approach in writing this series of papers will be to systematically illuminate on what it depends, and why.

#### Critical sub-topics in nanomaterial data curation

A paper will be developed for each of a number of sub-topic groups relevant to nano curation (Table 1). We acknowledge the vast scope of the topics as outlined in Table 1, each of which is complex and relevant to informatics approaches within many other fields. This is a dynamic initiative and the list is provided as a starting point; it may grow and/or change over time through community dialogue and the identification of topical areas that are in need of exploration and clarification. We may also choose to condense and rearrange subtopics, but the list below represents the primary ideas generated collectively by the NCIP NanoWG, and reiterated by the participation of nanomaterial data curation stakeholders (to be discussed below). The currently planned series of NDCI papers is scheduled for production over the next two years, with the first manuscript accepted for publication in the Beilstein Journal of Nanotechnology, the following three in preparation, and the final topic being scoped by a designated author team.

**Table 1:** NDCI curation sub-topics.

#	sub-topic area	planned focus

1	curation workflows	Addresses workflow aspects such as curation protocols for consuming data from primary literature as well as data transfers between repositories or between data customers and data consumers. Discusses mechanisms for both primary curation of data into repositories and interoperable sharing between resources. A direct comparison of officially documented and/or informally institutionalized curation protocols will provide a clear baseline and allow concrete discussion of next steps for protocol standardization. Also addresses a starting point for the workflows in terms of sourcing, including various approaches for identifying sources: active sourcing, where the data repository does the work (either automated or manual) of identifying data sources, or passive sourcing where the dataset owners are the agents that seek access to the repository.
2	data completeness and quality	Includes discussion of both data quality and data completeness. Completeness is a measure of the raw data, assays, processed data, or derived data. What are different ways data completeness could be defined, and are these completeness criteria shaped of the goals for the data being curated? High quality data could still be sparse or “incomplete”, so separately, what approaches are employed to define and evaluate data quality? This sub-topic encompasses issues such as precision, error, and sufficiency of meta-data for reproducibility. Are there differences when evaluating data quality captured from a database versus from the primary literature?
3	curation responsibilities	Covers curation responsibilities, including established and developing roles and division of curation labor and exploring the real challenges associated with quantity vs. quality of data entries. Curation training and performance expectations will also be addressed, as will the roles of other non-curators in defining the curation process (e.g. how might data “customers”, such as peer-reviewed journals, influence the process).
4	integration between databases and datasets	How do we define and operationalize integration between databases and datasets? What level of interoperability is required to support data integration in a way that supports various goals for comparison and analysis? Specific topics that can be challenges to interoperability will be discussed, for example, questions such as what is the primary key – the root or kernel that makes an individual record unique? Some infrastructures base the primary key on the nanomaterial, whether on the batch, the lot level, or just the product name. Others utilize a particular study or experiment as the basis around which the structure is oriented. This definition of a unique entry into a database is fundamental to the structure of the database, often differs between different resources, and greatly impacts how data are curated from a source. Finding ways to map across these differences in record definition will be an important consideration.
5	metadata	The way metadata are handled within a database and within data records is critical to every other nanotechnology data curation topic listed. For example, environmental and biological media characterizations are critical for interpretation as well as comparison of data. Temporal metadata are also key; how experimental and characterization timing is incorporated to data collection and infrastructure is integral to enabling reproducibility of data and to achieving functional interoperability between datasets.

In each paper, we will examine each of the sub-topics, identified in Table 1 following this consistent discussion structure:

1. Why this sub-topic is important and relevant to the understanding of nanomaterial data curation, and the subsequent functional interoperability of datasets.
2. How does the purpose of an individual nanomaterial data resource or curation effort (e.g., to inform product development, to identify data gaps for research prioritization) impact (i) the approaches to this aspect of curation and (ii) particular challenges involved with this aspect of curation?
3. What are established handling methods for this sub-topic in mature fields (e.g., biological data curation)?
4. What are key challenges specific to emerging materials/nanomaterials with regard to this sub-topic? Are there any specific use cases to illustrate these issues and make them tangible?
5. What are some recommendations for advancing nanomaterial data curation in support of functional interoperability between datasets and resources: (i) Opportunities to leverage existing nanoinformatics resources (e.g. ISA-TAB-nano) in addressing integration for this sub-topic, or reasons not to do so? (ii) Practical next steps for individual stakeholders or the community as a whole?

## Results and Discussion

For each sub-topic paper, information relevant to the discussion topics listed above will be gathered from a group of Stakeholder Liaisons who represent various organizations with activities related to curation of nanomaterial data. The role of the Stakeholder Liaisons will remain consistently defined throughout the NDCI series, but the make-up of the group is envisioned as dynamic. First, with increasing visibility of the project, it is the hope of the authors to gain more interest and widen participation in the Stakeholder Liaison group. While maximum retention will be sought for consistency and comparison across all topics, realistically the NDCI team realizes some individuals may choose to be involved in all papers within the series while others may elect to abstain from a given paper given interest or time constraints. In the interest of maximizing the scope of the baseline view of the nanocuration field, the NDCI will be inclusive of all Stakeholder Liaison responses. Our first step in this project was to identify these stakeholders through a series of inquires sent out by appropriate members of the NCIP NanoWG leadership team. Five organizations responded to our initial invitation recruiting Stakeholder Liaisons and provided answers to a set of foundational questions for this initial framing paper; their responses are presented in Tables 2–4 (see below). It is important to note that all Stakeholder Liaisons have been made explicitly aware that their names and institutions are associated with their responses to these questions, in an effort to foster a transparent discussion; all respondents were also provided the opportunity to review the final draft of this manuscript for as inclusive a process as possible. Several more have agreed to serve as Stakeholder Liaisons going forward on the other sub-topic papers, and we intend to continue expanding upon the initial group as this project moves forward. We will begin each sub-topic paper process by the NCIP NanoWG leadership team posing a set of questions to the Stakeholder Liaison group. A period of one month is allotted for response preparation, and the NDCI team has committed to circulating no more than one set of questions at a time to address the topics in series and to be mindful of the time and effort requirements placed on the Stakeholder Liaisons. As in this article, all stakeholder responses will be presented in the published articles to transparently represent the community perspectives; although as the liaison list grows, due to various limiting considerations of some participating organizations, decisions may be made to forego full liaison transparency in favor of being able to include the input of as broad as possible a swath of nanomaterial data stakeholders. Together, the responses provide a baseline snapshot of current practical experiences, and a range of views that will feed into a synthesized summary of recommendations addressing curation on behalf of the nanoinformatics community. The collection of this diverse and expanding group of stakeholder perspectives will foster development of preliminary recommendations for how to advance nanomaterial curation in principal and in practice, while identifying a community of practice in the process.

### Establishing a baseline of nanomaterial curation considerations

For the current article, the NCIP NanoWG leadership team established communication with individuals in the current nano-curation Stakeholder Liaison group and posed three fundamental questions:

1. Briefly describe the scope (goals and research questions) of your data curation efforts.
2. What do you believe are the major challenges in nanoscience/nanotechnology data curation?
3. Within your effort, what data (information) is necessary to directly compare nanomaterials and determine if they are the same material?

As expected, responses showed variety in both purpose (of the resource and the organizations represented) and scope. In response to the first question, the responses show that the purpose of curation encompasses efforts across the life cycle of nanomaterials and the life cycle of datasets generated about nanomaterials (Table 2). Some efforts focus on capturing data at the point of generation (academic or industrial research), and some focus on capturing data after its packaging and release in publications. Stakeholder representation from across the ENM-product life cycle presents an opportunity to identify and enable information hand-offs that facilitate targeted integration of nanomaterial data. The differences in curation scope will allow us to explore the extent to which curation practices need to be the same in order to enable data comparison. In addition, we may be able to identify whether or not there are drivers to integrate datasets between organizations with very specific and more general scopes.

**Table 2:** Liaison question #1.

liaison	affiliation	scope of data curation effort

Bill Zamboni	UNC	My research program at UNC is involved in the profiling and translational development of nanoparticle agents. My research program focuses on evaluating the pharmacokinetics (PK) and pharmacodynamics (PD) of nanoparticle agents in preclinical models and in patients. Specifically, we are involved in evaluating the factors that alter the function of the mononuclear phagocyte system (MPS) which then alters the PK and PD of nanoparticle agents in preclinical models and in patients. We have developed phenotypic probes of MPS function that predicts the PK and PD of nanoparticles in animals and patients. We are also developing a high throughput screen (HTS) of the interaction between nanoparticles and the MPS which predicts in vivo PK of the nanoparticles. The MPS HTS can be used to screen and select nanoparticles with high and low MPS uptake prior to going into in vivo studies. We are also evaluating how the MPS may be involved in the clearance and distribution of nanoparticles via capture (i.e. nanoparticle goes to the spleen and then is taken up by the MPS) and/or hijacking (i.e. the nanoparticle is taken up by the MPS cells in the blood and then delivered to tissues while inside the MPS cells).
Christoph Steinbach, Clarissa Marquardt	DaNa database NanoRA	The goal of our project is to provide impartial information and the real knowledge on safety aspects of (manmade) nanomaterials. DaNa in the acronym for DAtabase NAnomaterials but today we prefer talking about our Knowledgebase Nanomaterials and that describes our goals very well: We try to separate publications which are suitable for assessment of safety aspects of nanomaterials from those who are not suitable. So we try to collect not only arbitrary data but scientifically proven knowledge. The need to perform such kind of assessment is documented e.g., in a publication by Hristozov et al. [11].
Marina (Nina) Vance	nanotechnology Consumer Products Inventory	Our curation effort is centered on the nanotechnology Consumer Products Inventory (CPI). The CPI was developed by the Woodrow Wilson International Center of Scholars in 2005 and it is currently the most comprehensive listing of consumer products that contain or claim to contain nanomaterials. The main goal of the CPI is to document the way in which nanotechnology is entering the consumer market. Specifically, we want to provide the science and regulatory communities, as well as consumers, with current and accurate information about nano-enabled consumer products and the nanomaterials they contain.
Christine Ogilvie Hendren	CEINT NIKC (Center for Environmental Implications of NanoTechnology NanoInformatics Knowledge Commons)	Our curation effort is centered around interrogating the data gathered from across the Center for Environmental Implications of Nanotechnology along with comparative literature from throughout the field external to the center. Though our controlled material sourcing has created a rich integrated dataset as a starting point, we have a wide range of data types and fields, representing our focus on complex environmental interactions and transformations as well as impacts across a biological continuum and including ecosystem-wide measures. Our central research goals driving the data integration process are to 1) Probe mechanistic relationships between material and system properties and their combined effects on nanomaterial fate and effect in the environment, 2) Organize our disparate data to provide directional guidance to risk assessors even prior to achieving goal 1, and 3) Test our hypotheses that a amassing data on a small number of semi-empirical functional assays measurements will allow us to further goals 1 and 2. Beyond supporting CEINT mission-focused research questions, two key goals of our data integration project are to build a cyberinfrastructure that captures the data in a way that enables reproducibility and quality control down the road, and to ultimately develop associated tools to involve researchers in self-curation of their data so they can shorten the curation timeline and realize the benefits of analyzing their data together with other comparable datasets.
Julio Cesar Facelli, David Eugene Jones	NanoSifter (University of Utah)	The purpose of the NanoSifter project here at the University of Utah is to create a natural language processing (NLP) tool which is capable of extracting nanoparticle data associated to nanoparticle properties directly from the primary literature. Currently, the tool can extract data associated to hydrodynamic diameter, particle diameter, molecular weight, zeta potential, cytotoxicity, IC_50_, cell viability, encapsulation efficiency, loading efficiency, and transfection efficiency. We plan to expand the information that NanoSifter can extract, while also improving the precision, recall, and f-measure of this tool.

The stakeholder responses to the second question we posed on challenges to curation (Table 3) include aspects of every sub-topic area to be addressed within this project, including social aspects, such as reluctance to share, data quality issues, ontology development and adoption decisions, and a simple lack of data. Other issues listed pertained to larger epistemological issues pervasive throughout the field of nano science. These included uncertainty about which material and system parameters are appropriate for predicting material behavior and interaction; and the struggle to make near-term decisions based on emerging science.

**Table 3:** Liaison question #2.

liaison	affiliation	major challenges to curation of nanomaterial data

Bill Zamboni	UNC	The complexity and high variability nature of MPS function in animal models and patients which results in high PK and PD variability of nanoparticles. The current inability to predict nanoparticle PK and PD in vivo based on standard critical micelle concentration (CMC)-like measurements (e.g., size and charge). The need to evaluate the interaction between the MPS and nanoparticles early in development and even before going into in vivo studies.
Christoph Steinbach, Clarissa Marquardt	DaNa database NanoRA	We think we are taking care of one of the most important challenges in nanomaterials data curation: separating valid from invalid data. In this regard, the major challenge is to gain information on the identity of a nanomaterial in a given study, which involves a careful physical-chemical characterization of a nanomaterial. Most of the data we consider invalid has a lack of information on material properties, which also hampers comparability of studies. Moreover, the collection of standard operating protocols (SOPs) or harmonized protocols for nanotox-testing is the second important challenge we want to address within the next four years. From a more information technological point of view, the development of suitable data models and adequate ontological structures to support next generation electronic infrastructures is another challenge.
Marina (Nina) Vance	Nanotechnology Consumer Products Inventory	One major challenge we face is a general lack of support from the nanotechnology industry. Secrecy is inherent to the product development strategy of most companies, which makes it very difficult to provide a detailed characterization of industrial nanomaterials. A potential contributing factor to this problem, which applies specifically to the CPI, is a fear that association to the CPI may negatively affect the image of the consumer products. Another challenge we face in curating the CPI is keeping it up to date with the fluidity of the consumer market. Consumer products come and go daily, their names and models change over time, as do their companies’ websites. To attempt to tackle this issue, we have added crowdsourcing capabilities to the CPI, so that interested consumers, manufacturers, or researchers can enter new data or suggest edits to any entry. Now, our main challenge is to catalyze the participation of the CPI contributors.
Christine Ogilvie Hendren	CEINT NIKC (Center for Environmental Implications of NanoTechnology NanoInformatics Knowledge Commons)	Absence of established data-sharing protocols for existing measurement techniques (not to mention those that are currently being developed). Complexity of the interactions of nanomaterials in the environment, and large numbers of influential parameters governing transformations. Wide range of variety in systems studied and particular parameters reported in those systems. How time points are handled with respect to explaining when materials were characterized, measured along the trajectory of a long-term experiment is a challenge; this gets back to our driving goal of creating a database that supports reproducibility and multi-study comparison.
Julio Cesar Facelli, David Eugene Jones	NanoSifter (University of Utah)	In my opinion, there are a number of major challenges in nanoscience/nanotechnology data curation. The first is developing standards and protocols to report data in the literature which the nanoscience/nanotechnology community adheres to and follows. There are so many different ways that properties of nanoparticles can be reported in the literature, which makes the retrieval of such information quite cumbersome. Another major challenge is further development of the nanoparticle ontology (NPO) to add more functionality, metadata, and relationships to the ontology.

The stakeholder responses to the open-ended question on comparison of nanomaterials all honed in on the critical question begged by asking what materials are the same: what do we mean by “sameness”? Similar definitional questions arose around curation resource purpose (Table 4).

**Table 4:** Liaison Question #3.

liaison	affiliation	data deemed necessary for nanomaterial comparison

Bill Zamboni	UNC	The need to be able to evaluate encapsulated/conjugated and released drug as part of formulation development and as part of in vivo PK studies. The need to evaluate biodistribution differences to tumor, tissues and the MPS. The need to evaluate the bi-directional interaction between nanoparticles and the MPS.
Christoph Steinbach, Clarissa Marquardt	DaNa database NanoRA	A very good question which is extremely hard to answer: What does “same material” mean, not only from the informational point of view but also from the other side, the definition of “same material”? Which set of parameters do you need? Even if you change the size or shape of a particle totally different behavior can be achieved. We have developed a set of criteria (see http://www.nanopartikel.info/files/methodik/DaNa-Literature-Criteria-Checklist_Methodology.pdf) which need to be fulfilled that we accept a certain publication as “knowledge” in the meaning described in the answer to the first question. Here we also describe the material characterization criteria. In fact we are absolutely aware that this does not make finally sure, that we are always talking of the “same” material, but for our purposes it’s enough. We think that a lot of further research is necessary to determine the right “same material” parameters. Furthermore the comparability in nano-sciences does not end with the “same” material as it is shown in certain round robin experiments [12–13]. Does it help when you assume to have the same material and the following experiments show different results because of other factors? I do not know if that leads to a better solution: Perhaps some kind mathematical probability that tells us *x* parameters (out of *y* parameters which can be determined with today’s characterization methods) of one substance are the same for another. The higher the number of same parameters the higher the probability the two substances are the “same”?
Marina (Nina) Vance	Nanotechnology Consumer Products Inventory	Within the CPI, it is very difficult to determine if a nanomaterial present in two or more products is, in fact, the same. We can group nanomaterials of the same composition together, but without a detailed description from the manufacturer, that would be impossible. In order to directly compare nanomaterials within consumer products, we would need, in the very least, the following: Composition, Shape, Size, Composition of coatings, Crystallinity
Christine Ogilvie Hendren	CEINT NIKC (Center for Environmental Implications of NanoTechnology NanoInformatics Knowledge Commons)	This depends on the level of granularity in the comparison. We believe that in order to support comparison and analysis in support of our research goals (elucidate mechanisms governing nanomaterial behavior and translate this into forecasts of risk), what is absolutely required are intrinsic characteristics of the nanomaterial, the surrounding system characteristics (e.g., be the system lab controlled, environmental media, biological systems), and system-dependent or "extrinsic" material characteristics. Only when all of these aspects, and their appropriate corresponding metadata describing preparation and testing protocols, are consistently reported can we know that direct comparison of two datasets is possible.
Julio Cesar Facelli, David Eugene Jones	NanoSifter (University of Utah)	The data (information) that is most necessary to directly compare nanomaterials and determine if they are the same material are the molecular descriptors and biochemical activity of the nanomaterials. The molecular descriptors (e.g., molecular weight, hydrodynamic diameter) and biochemical activity (e.g., cytotoxicity, cell viability, transfection efficiency) of the nanomaterials can be used by data mining and machine learning methods to compare materials and determine their similarity if the materials are discrete compounds. If the materials are not discrete compounds (i.e., polymers), properties such as molecular weight distribution and polydispersity will be the properties to assess for comparison of materials.

From these initial framing questions alone, it is clear that in order to make progress in integrating data through consistent nano curation processes, and to achieve functional interoperability that will render efforts to establish nanoinformatics fruitful, the nanomaterial community will have to maintain a focus on the need for purpose-based integration. Therefore through interaction with stakeholder liaison that will follow this inaugural publication, and the synthesis of their input, we will distill the recommended tenets of nanomaterial data curation both in terms of baseline requirements for all nanoinformatics activities as well as for a range of purposes.

The experience to date in the NCIP NanoWG and in assembling the NDCI has already begun addressing the third NDCI goal of facilitating the interdisciplinary and trans-sector collaborations that we believe will be critical ingredients in successful advancement of nanoinformatics efforts. The team-writing experience within the author teams of the NDCI topic papers includes promising aspects that can foster collaborations. For each topic paper, a group of self-selected NCIP NanoWG members are volunteering to lead the topic, assembling author groups that, in the case of the four papers already being undertaken, often consist of people who have never collaborated or published together prior, and soliciting the broad input provided by Stakeholder Liaisons across the nanomaterial data community. New connections are being made between individuals and organizations, and for each topic these new teams are working through the available literature across a variety of academic disciplines, synthesizing the baseline input from Stakeholder Liaisons, and shaping recommendations and future questions for the consideration of the growing nanoinformatics field. Though there are not direct Stakeholder Liaison interactions planned as part of the NDCI, the transparency and sharing of their responses through the NDCI series will offer fertile ground for potential communication and collaboration between like or complimentary groups in future efforts. Lastly, the recommendations emerging from the NDCI series will no doubt include suggestions on opportunities regarding the potential for linkages and collaborations.

We welcome input from the nanomaterial community on the approach for the project laid out in this article and encourage continued feedback as the effort moves forward, including via participation from growing list of nanomaterial data stakeholders. Interested community members can share feedback or join the NCIP by visiting to https://nciphub.org/, and can learn more about the NDIC in particular by visiting https://nciphub.org/groups/nanotechnologydatacurationinterestgroup/wiki/MainPage.
